# The effect of balance exercises on central sensitization in patients with knee osteoarthritis

**DOI:** 10.1007/s00296-024-05550-3

**Published:** 2024-03-16

**Authors:** Emre Tirasci, Tunay Sarpel, Ilke Coskun Benlidayi, Volkan Deniz

**Affiliations:** 1 Department of Physical Medicine and Rehabilitation, Faculty of Medicine, Cukurova University, Adana, Türkiye; 2 Department of Physiotherapy and Rehabilitation, Faculty of Health Sciences, Tarsus University, Mersin, Türkiye

**Keywords:** Exercise, Osteoarthritis, Knee, Central sensitization, Postural balance

## Abstract

The aim of this study was to evaluate the effectiveness of balance exercises on functional status, pain, balance, and central sensitization in patients with knee osteoarthritis (OA). Patients diagnosed with bilateral Kellgren–Lawrence grade ≥ 2 primary knee OA and associated central sensitization were included in the study. Patients were randomized into two groups. Both groups were provided with verbal and written information on knee OA. In addition, the intervention group received a supervised balance exercise program for 6 weeks, 3 days a week on alternating days. The outcome measures were the changes in the Central Sensitization Inventory (CSI), Visual Analog Scale (VAS) pain, the Western Ontario and McMaster Universities Osteoarthritis Index (WOMAC), Berg Balance Scale, and Y Balance Test. Evaluations were performed at baseline, immediately after treatment (6th week) and at 12th week. The study included 40 patients, 20 patients in each group. At the end of the treatment period (6th week), the improvement in CSI score, WOMAC pain, WOMAC physical function, WOMAC total score, Y Balance Test scores, and VAS pain during activity was significantly greater in the intervention group than that in the control group (*p* < 0.001). Regarding the changes from baseline to the 12th week, the intervention group experienced greater improvement in most of the outcome measures. Yet, the change in WOMAC pain score, Berg Balance Scale score, and VAS pain at rest was similar between the study groups (*p* = 0.05, *p* = 0.257, and *p* = 0.385, respectively). A two-model multiple linear regression analysis revealed that the changes in VAS pain (during activity) after the treatment and at follow-up [(*p* = 0.004, adjusted *R*^2^: 0.346) and (*p* = 0.002, adjusted R^2^: 0.391), respectively], as well as changes in WOMAC pain from baseline to follow-up (*p* = 0.020, Δ*R*^2^ = 0.245) significantly affected central sensitization. However, changes in *Y* Balance Test and WOMAC total scores did not appear to have a significant impact on the improvement in central sensitization (*p* > 0.05). Balance exercises may provide improvement in central sensitization, functional status, and dynamic balance among patients with knee OA. The improvement in central sensitization depends mostly on the pain relief effect of balance exercises.

## Introduction

Knee osteoarthritis (OA) is a chronic degenerative disease in which mechanical and biochemical factors play a significant role. Morphological changes are seen in joint structures, including the cartilage, subchondral bone, and synovial membrane [1]. Pain experienced in knee OA is attributed to these structural changes. Yet, psychosocial and neurophysiological factors also play a role in this multifactorial phenomenon [2]. OA-related pain was considered pure nociceptive pain in the past. It is now seen as a complex phenomenon in which nociceptive and neuropathic components coexist; peripheral and central sensitization pathways play a role [3]. Central sensitization, known as the increased response of nociceptive neurons in the central nervous system to normal or subthreshold afferent stimuli [4], should definitely be considered in patients with OA. Several studies aiming to control central sensitization and improve pain in OA have been conducted so far [5–7]. However, due to insufficient literature data, management guidelines on central sensitization could not be developed. Although pharmacological treatment methods such as opioids, serotonin, and noradrenaline reuptake inhibitors [5], as well as non-pharmacological treatment methods such as pain neuroscience education, cognitive behavioral therapy [6], manual therapy, and electrotherapy [8] are applied, sufficient efficacy is often not achieved. Exercise is often used as a fundamental component in the management of OA-related pain. Although the benefits of exercise in knee OA have been clearly documented, whether it has beneficial effects on central sensitization is still being determined [7]. A recently published systematic review emphasized that the evidence that exercise approaches lead to an improvement in central sensitization in patients with knee OA patients is still insufficient [9]. Proprioceptive defects and weakness of the quadriceps muscle can cause postural control and balance deterioration in patients with knee OA [10, 11]. Pain sensation caused by joint inflammation prevents the formation of afferent proprioceptive stimuli and causes instability that limits the individual’s functional status [12, 13]. Therefore, management of balance loss is vital in patients with knee OA. There are different results in the literature regarding the effects of exercise regimens on balance in patients with knee OA. In general, results revealed the beneficial effects of exercising on balance and postural control in knee OA [14–16]. Yet, some studies reported no significant improvement in the exercising groups compared to the control groups [17, 18]. Given the conflicting results in the literature, the effects of exercising on static/dynamic balance in patients with knee OA need to be further studied. Moreover, as a relatively new topic, the potential benefits of balance exercises in terms of central sensitization should be investigated. In light of the background mentioned above and gaps in the literature, the main aim of this study was to examine the effect of balance exercises on central sensitization in patients with knee OA. The secondary aim was to investigate the potential impact of balance exercises on pain, balance, and functional status.

## Patients and methods

### Design of the study

A randomized, controlled, single-blind study design was applied. The Ethics Committee of Cukurova University Faculty of Medicine approved the study (Date: 14.02.2020, decision no: 96), and the study protocol was registered to clinicaltrials.gov (NCT04308967). The study was conducted at a tertiary university hospital between March 2020 and April 2021. Written and verbal information about the protocol of the study was given to all participants, and written informed consent was obtained.

### Participants

**Fig. 1:**
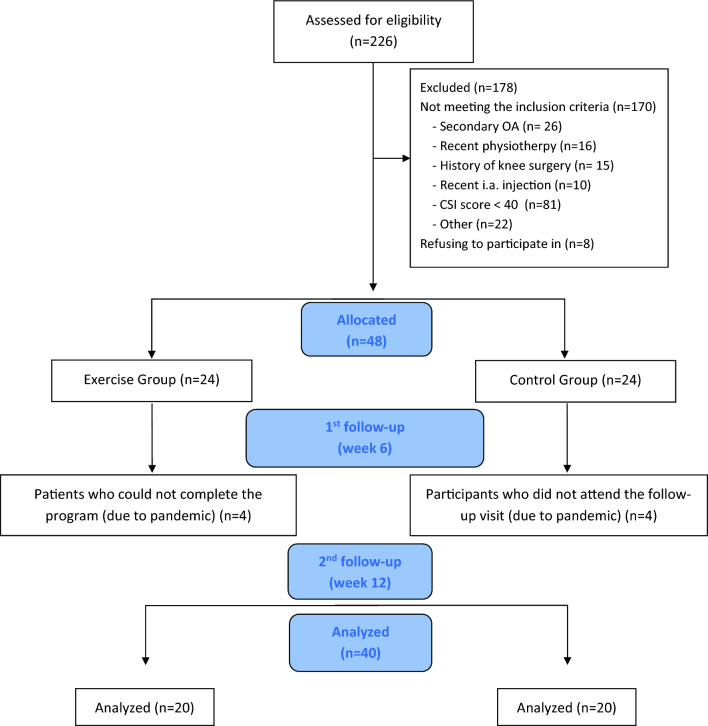
Flowchart of the study

Patients who met all of the following inclusion criteria were included in the study: (i) diagnosed with primary knee OA based on the American College of Rheumatology criteria [19], (ii) having bilateral grade 2 or higher knee OA according to the Kellgren and Lawrence (KL) radiological grading system [20], and (iii) having a central sensitization score of 40 or higher according to the Central Sensitization Inventory (CSI) [21]. Patients who [i] were diagnosed with secondary knee OA, [ii] had at least one of the diseases in the B part of the CSI [21], (iii) had severe cognitive impairment, (iv) had a history of knee surgery, (v) received physiotherapy to the knee in the last 6 months, and (vi) had any injections to the knee joint during the previous 6 months were excluded. Patients were assigned to the treatment and control groups via the computerized randomization method by the research physiotherapist who did not participate in the evaluation process (Fig. 1). The evaluation of the participants was performed by a physiatrist who was blinded to the groups.

### Treatment protocol

**Fig. 2:**
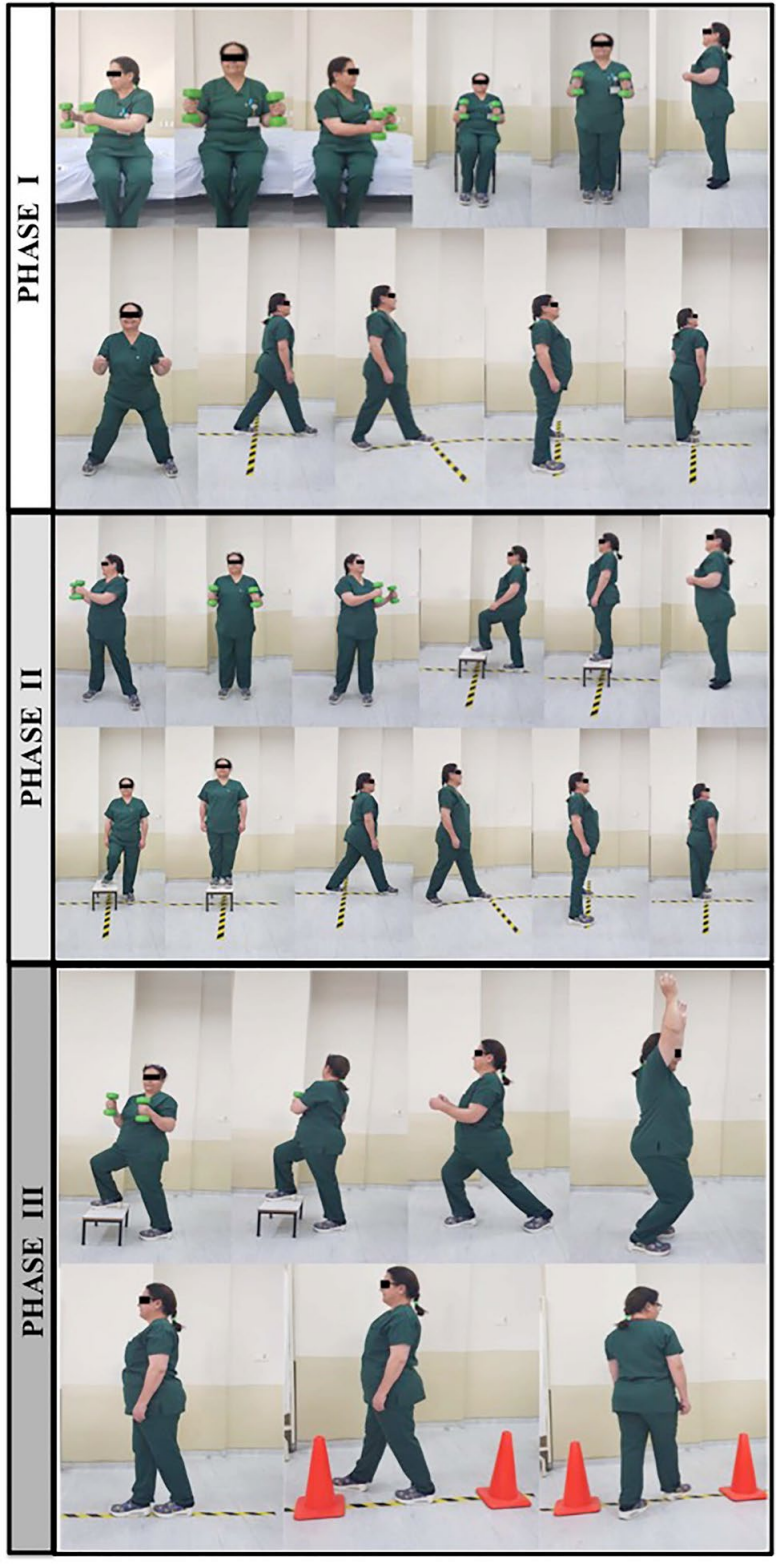
Balance exercise program

The treatment group received a balance exercise program (Fig. 2), which consisted of three phases (each phase for 2 weeks), 3 days a week on alternating days for a total of 6 weeks. An exercise program, which was proven to be effective previously, was adopted, modified, and performed under the supervision of a physiotherapist [22]. The exercises were performed in three sets of eight to twelve repetitions. A five-minute aerobic warm-up period was applied before each session. Phase I of the program (0–2 weeks) included the following exercises: (i) in sitting position, holding 1 kg weight in hands and performing trunk rotation, (ii) standing up from sitting while holding the weights, iii) in standing position, rising on toes, (iv) side stepping aside while knees are flexed slightly, and (v) stepping each side on a plus ( +) sign formed by sticky bands on the floor. Phase II of the program (2–4 weeks) included the following exercises: (i) in standing position, holding 1 kg weight in hands and performing trunk rotation, (ii) climbing one stair up, (iii) rising on toes and walking slowly, (iv) stepping sideways onto step, and (v) stepping each side on a plus ( +) sign formed by sticky bands on the floor. Phase III of the program (4–6 weeks) included the following exercises: (i) starting from standing position, stepping one stair up while holding 1 kg weight in hands and performing trunk rotation, (ii) stepping forward and lowering into lunge, (iii) hopping while knees are flexed slightly, (iv) Tandem walk, and (v) 8-walk around cones [22] (Fig. 2).

All patients in the treatment and control groups were informed about knee OA and the modifications that should be made in daily living activities to reduce the symptoms. Besides, a brief pain neuroscience education and a written brochure were given to the participants. The patients were allowed to use paracetamol (acetaminophen) when needed without exceeding 2 g/day.

### Evaluations

Demographic characteristics such as age (years), gender, body height (cm), body weight (kg), and body mass index (BMI) of the patients were recorded. The assessment tools/questionnaires included the CSI, Visual Analog Scale (VAS), Berg Balance Scale, Y Balance Test, and Western Ontario and McMaster Universities Osteoarthritis Index (WOMAC). The patients were evaluated by the same investigator three times: before the treatment (t0), at the end of the treatment (week 6) (t1), and at the 12th week (t2).

#### Central Sensitization Inventory (CSI)

Central sensitization was evaluated by the CSI [21]. This inventory has moderate reliability in patients who develop central sensitization due to knee OA [23]. The CSI consists of 2 parts, part A and part B. Part A contains 25 items related to central sensitization symptoms, scored on a 4-point Likert scale. The total score in section A ranges from 0 to 100 points. A score of 40 and above indicates central sensitization (sensitivity: 81% and specificity: 75%). Part B evaluates whether the patient had been diagnosed with another disease in the family of central sensitization syndromes by any physician. A high score obtained by CSI indicates severe central sensitization [21].

#### Visual Analog Scale (VAS)

Pain intensity at rest and pain during activity were evaluated by VAS. One end of the scale with a length of 10 cm represented “0 = no pain” and the other end represented “10 = most severe pain.” The value found by measuring the distance from the place marked by the patient to the “0 = no pain” point represented pain intensity [24].

#### Berg Balance Scale

Berg Balance Scale was used to evaluate functional balance [25]. Berg Balance Scale has high validity [intraclass correlation coefficient (ICC) = 0.95] in patients with knee OA [26]. The Berg Balance Scale is a 14-item test. A 4-point Likert scale is used for each item. The person gets a score between 0 and 56. Points between 0 and 20 represent “high fall risk”; 21–40, “medium fall risk”; and ≥ 41, “low fall risk”[25].

#### Y Balance Test

*Y* Balance Test, which requires strength, flexibility, and proprioception, was used to evaluate dynamic balance. This test includes extensions in the anterior, posterolateral, and posteromedial directions. The patient stands on one foot at the midpoint of the test setup. Then, the patient touches the anterior, posteromedial, and posterolateral directions with the other foot while maintaining balance with the toe tip. In all directions, the test was performed three times. The average is recorded in centimeters. The score was obtained by taking the average of three elongations for each direction, dividing this average by the leg length, and multiplying by 100 [27]. It has high reliability with median ICC values for intra-rater reliability being 0.88 (0.84–0.93), 0.90 (0.68–0.94), and 0.88 (0.85–0.94) for the anterior, posterolateral, and posteromedial directions, respectively [28].

#### Western Ontario and McMaster Universities Osteoarthritis Index (WOMAC)

WOMAC was used to assess physical function, knee pain, and stiffness. This questionnaire, which consists of 24 questions and three parts (pain, stiffness, physical function), also has good validity and reliability in patients with knee OA [29]. The total WOMAC score ranges from 0 to 30, with high scores indicating severe loss of function, pain, and stiffness. The minimum clinically significant difference is 1.33 points.

### Statistical analysis

A per-protocol analysis was conducted, and data of the participants who partook in three assessments were analyzed. Statistical analysis was performed using the IBM^®^ SPSS^®^ Statistics for Windows, version 23.0 software (IBM Corp. Armonk, NY, USA). Visual methods (histograms and probability plots) and the Shapiro–Wilk test were performed to test the normality of data. Descriptive data were expressed in mean and standard deviation (SD) and number/frequency. To analyze within-group changes and between-group differences, a two-way repeated measures of analysis of variance (ANOVA) test [time × group (within-subject × between-subject)] were applied. A *p* value < 0.05 was considered statistically significant. Cohen’s *d* was calculated for the effect size of the significant difference. For within-group pairwise comparisons of delta values, a Bonferroni correction was applied. *p* values < 0.017 were considered statistically significant. A two-model multiple linear regression analysis was performed to determine the underlying factor(s) through which balance exercises improved central sensitization. In this analysis, change in VAS pain (during activity) constituted the first model as an independent variable. Delta values of Y Balance Test and WOMAC pain, WOMAC physical function, and WOMAC total scores were included in the second model. A *p* value < 0.05 was regarded as statistically significant. The required sample size was calculated using the G*Power^®^ program (Heinrich Heine Universität Düsseldorf, Düsseldorf, Germany). ANOVA repeated measures and within-between interactions were used in the system. Considering the study performed by Lluch et al. [6], the effect size was determined at 0.252. The correlation between repeated measurements assumed was 0.5. Considering three measures in two study groups, the sphericity correction was determined at 1.0. The statistical power was set as 0.95 and the alpha level as 0.05. The sample size was estimated as 44 participants. Considering a possible loss to follow-up of up to 10%, 48 patients (*n* = 24 in each group) were recruited.

## Results

Of the 226 patients assessed for eligibility, 48 who met the inclusion criteria were assigned to the treatment (*n* = 24) and control groups (*n* = 24). The data of 40 patients (treatment group = 20, control group = 20) who participated in the exercise program at a rate of 85% or more and whose data were collected in all three evaluations were analyzed (Fig. 1). At the beginning of the study, no statistically significant difference was observed between the treatment group and the control group in terms of gender, KL grade, age, height, weight, and BMI values (*p* > 0.05 for all) (Table 1). The results revealed a significant group*time interaction in the CSI score (*F* = 42.143; *p* < 0.001; *η*_p_^2^ = 0.526), VAS pain at rest (*F* = 19.144; *p* < 0.001; *η*_p_^2^ = 0.335), *Y* Balance Test total scores (for left leg: *F* = 73.174; *p* < 0.001; *η*_p_^2^ = 0.658, for right leg: *F* = 74.395; *p* < 0.001; *η*_p_^2^ = 0.662), and WOMAC total score (*F* = 16.585; *p* < 0.001; *η*_p_^2^ = 0.304). There was no significant interaction in VAS pain at rest (*F* = 2.731; *p* = 0.072; *η*_p_^2^ = 0.067) and Berg Balance Scale score (*F* = 1.974; *p* = 0.168; *η*_p_^2^ = 0.049). When the time-dependent changes in the treatment group (in-group evaluation) were examined, there was a significant difference in all tested parameters. In the control group, significant improvement was observed in VAS pain, Berg Balance Scale score, left leg *Y* balance anterior, posteromedial and total scores, posteromedial and total scores for the right leg, WOMAC stiffness, WOMAC physical function, and WOMAC total scores (Table 2). When the changes (delta) in clinical parameters from baseline (t0) to the 6th week (t1) were compared between the two groups, improvement in the CSI score, VAS pain during activity score, the Y Balance Test scores for the left and right legs in all aspects, WOMAC pain, WOMAC physical function, and WOMAC total scores were significantly higher in the treatment group compared to that in the control group (Table 3). When the changes in clinical parameters (delta values) from baseline (t0) to the 12th week (t2) were analyzed, it was determined that the treatment group was superior to the control group in the improvement of all parameters except for VAS pain at rest, WOMAC pain, and Berg Balance Scale scores (Table 3).

**Table 1:** Comparison of the demographic variables and KL grades between treatment and control groups

	Treatment group (*n* = 20)	Control group (*n* = 20)	*p*
Age (years) (mean ± SD) (min-max)	54.3 ± 8.6 (41–71)	56.0 ± 9.9 (40–73)	0.567
Body height (cm) (mean ± SD) (min-max)	162.1 ± 6.7 (151–176)	158.0 ± 6.0 (145–167)	0.058
Body weight (kg) (mean ± SD) (min-max)	80.7 ± 14.6 (55–110)	79.6 ± 10.5 (60–100)	0.776
BMI (kg/m^2^) (mean ± SD) (min-max)	30.7 ± 5.0 (22.6–40.4)	32.0 ± 5.2 (23.4–41.6)	0.395
Sex n (%)			
Female	17 (85.0)	17 (85.0)	> 0.999
Male	3 (15.0)	3 (15.0)	
KL grade (%)			
Grade I	5 (25)	4 (20)	
Grade II	13 (65)	14 (70)	0.929
Grade III	2 (10)	2 (10)	

*BMI* body mass index, *KL* Kellgren–Lawrence

**Table 2:** Within-group comparison of changes in clinical parameters by time

	Group	Pretreatment (t0) (mean ± SD)	Posttreatment (t1) (mean ± SD)	12th week (t2) (mean ± SD)	*p*_t0-t1-t2_	*p*_t0-t1_	*p*_t0-t2_	*p*_t1-t2_
CSI score	Treatment	59.8 5.6	50.6 ± 5.1	48.7 ± 5.9	< 0.001	< 0.001	< 0.001	0.017
	Control	56.9 ± 6.6	55.5 ± 7.4	54.9 ± 7.7	0.104	0.113	0.098	0.377
VAS pain at rest	Treatment	3.1 ± 1.0	2.1 ± 0.7	1.8 ± 0.8	< 0.001	< 0.001	< 0.001	0.030
	Control	3.3 ± 1.0	2.6 ± 0.9	2.6 ± 0.9	0,003	0.002	0.005	0.564
VAS pain during activity	Treatment	8.0 ± 0.7	5.5 ± 0.9	5.2 ± 1.0	< 0.001	< 0.001	< 0.001	0.092
	Control	7.6 ± 1.0	6.8 ± 1.0	6.6 ± 1.6	0,003	0.004	0.004	0.102
Berg Balance Scale score	Treatment	54.2 ± 1.4	55.1 ± 0.8	55.1 ± 0.8	< 0.001	0.002	0.002	> 0.999
	Control	53.9 ± 1.3	54.4 ± 1.1	54.4 ± 1.1	0.001	0.008	0.008	> 0.999
Y Balance Test								
Left leg								
Anterior	Treatment	101.9 ± 4.9	104.9 ± 4.9	105.9 ± 5.2	< 0.001	< 0.001	< 0.001	0.001
	Control	101.9 ± 7.0	102.6 ± 7.2	102.8 ± 7.3	0.001	0.003	0.002	0.144
Posteromedial	Treatment	104.2 ± 4.2	107.0 ± 4.3	107.7 ± 4.1	< 0.001	< 0.001	< 0.001	0.006
	Control	103.2 ± 6.9	103.5 ± 6.5	103.6 ± 6.8	0.032	0.262	0.058	0.990
Posterolateral	Treatment	96.3 ± 4.5	98.9 ± 4.6	99.1 ± 4.9	< 0.001	< 0.001	< 0.001	0.063
	Control	96.3 ± 6.9	96.2 ± 7.1	96.4 ± 7.0	0.574	> 0.999	> 0.999	0.505
Total	Treatment	100.8 ± 4.3	103.6 ± 4.4	104.2 ± 4.6	< 0.001	< 0.001	< 0.001	< 0.001
	Control	100.4 ± 6.8	100.8 ± 6.9	100.9 ± 6.8	0.001	0.001	0.001	0.407
Right leg								
Anterior	Treatment	103.2 ± 4.4	106.3 ± 4.4	106.8 ± 4.5	< 0.001	< 0.001	< 0.001	0.071
	Control	103.8 ± 7.3	103.9 ± 7.2	104.2 ± 7.2	0.158	> 0.999	0.367	0.127
Posteromedial	Treatment	105.1 ± 3.9	108.3 ± 4.3	108.4 ± 4.2	< 0.001	< 0.001	< 0.001	0.594
	Control	104.3 ± 7.4	104.5 ± 7.4	104.8 ± 7.4	0.031	0.314	0.093	0.315
Posterolateral	Treatment	97.3 ± 4.8	99.8 ± 4.9	99.9 ± 5.0	< 0.001	< 0.001	< 0.001	0.515
	Control	97.0 ± 7.4	97.4 ± 7.1	97.5 ± 7.1	0.070	0.396	0.145	0.945
﻿Total	Treatment	101.9 ± 4.1	104.8 ± 4.2	105.0 ± 4.3	< 0.001	< 0.001	< 0.001	0.076
	Control	101.7 ± 7.3	101.9 ± 7.1	102.7 ± 7.1	0.029	0.455	0.066	0.051
WOMAC								
Pain	Treatment	6.1 ± 1.1	4.5 ± 0.8	4.0 ± 0.9	0.001	< 0.001	< 0.001	0.003
	Control	5.4 ± 0.8	5.0 ± 1.1	4.9 ± 1.3	0.137	0.131	0.174	0.965
Stiffness	Treatment	5.4 ± 1.0	3.8 ± 1.3	3.5 ± 1.0	< 0.001	< 0.001	< 0.001	0.013
	Control	5.3 ± 1.2	4.3 ± 1.3	4.3 ± 1.3	< 0.001	0.001	0.001	> 0.999
Physical function	Treatment	6.0 ± 1.0	4.8 ± 0.9	4.5 ± 0.9	< 0.001	< 0.001	< 0.001	0.004
	Control	5.9 ± 0.9	5.5 ± 1.1	5.4 ± 1.1	0.004	0.015	0.016	0.229
Total	Treatment	17.6 ± 2.7	13.2 ± 2.4	12.1 ± 2.3	< 0.001	< 0.001	< 0.001	< 0.001
	Control	16.8 ± 2.5	14.9 ± 3.2	14.7 ± 3.4	0.001	0.003	0.004	0.246

*CSI* Central Sensitization Inventory, *VAS* visual analog scale (VAS), *WOMAC* Western Ontario and McMaster Universities Osteoarthritis Index

*p* < 0.017 was considered statistically significant for pairwise comparisons

**Table 3:** Between-group comparison of changes in clinical parameters from baseline (t0) to the 6th week (t1) and from baseline (t0) to the 12th week

	Posttreatment (t1)–Pretreatment (t0)				12th week (*t*2)–Pretreatment (t0)			
	Treatment group (mean ± SD)	Control group (mean ± SD)	Effect size (d)	*p*	Treatment group (mean ± SD)	Control group (mean ± SD)	Effect size (d)	*p*
CSI score	− 9.2 ± 3.6	− 1.4 ± 2.7	0.84	< 0.001	− 11.1 ± 4.7	− 2.0 ± 2.8	2.31	< 0.001
VAS pain at rest	− 1.0 ± 0.7	− 0.7 ± 0.7	−	0.078	− 1.8 ± 0.8	− 1.3 ± 1.0	−	0.385
VAS pain during activity	− 2.5 ± 1.1	− 0.8 ± 1.0	1.81	< 0.001	− 2.9 ± 1.3	− 1.1 ± 1.3	1.48	< 0.001
Berg Balance Scale score	0.9 ± 1.0	0.5 ± 0.6	−	0.257	0.9 ± 1.1	0.5 ± 0.6	−	0.257
Y Balance Test								
Left leg								
Anterior	3.0 ± 1.4	0.8 ± 0.7	1.95	< 0.001	4.0 ± 1.9	0.9 ± 0.7	2.12	< 0.001
Posteromedial	2.8 ± 1.5	0.4 ± 0.8	1.96	< 0.001	3.4 ± 1.4	0.4 ± 0.7	2.66	< 0.001
Posterolateral	2.7 ± 1.7	0.0 ± 0.9	1.95	< 0.001	2.9 ± 1.3	0.1 ± 0.1	2.90	< 0.001
Total	2.8 ± 1.1	0.4 ± 0.4	1.96	< 0.001	3.4 ± 1.3	0.5 ± 0.4	2.96	< 0.001
Right leg								
Anterior	3.1 ± 1.5	0.2 ± 0.9	2.34	< 0.001	3.6 ± 1.4	0.4 ± 1.1	2.49	< 0.001
Posteromedial	3.2 ± 1.2	0.3 ± 0.6	3.00	< 0.001	3.2 ± 1.3	0.5 ± 0.9	2.37	< 0.001
Posterolateral	2.5 ± 1.1	0.3 ± 0.9	2.15	< 0.001	2.6 ± 1.5	0.4 ± 0.9	1.74	< 0.001
Total	3.0 ± 1.1	0.2 ± 0.6	3.10	< 0.001	3.2 ± 1.1	0.4 ± 0.8	2.85	< 0.001
WOMAC								
Pain	− 1.6 ± 0.7	− 0.5 ± 0.9	−	< 0.001	− 0.5 ± 0.5	− 0.1 ± 0.5	−	0.05
Stiffness	− 1.5 ± 1.0	− 1.0 ± 1.0	0.50	0.098	− 1.9 ± 1.0	− 1.0 ± 1.0	2.84	0.014
Physical function	− 1.2 ± 0.6	− 0.4 ± 0.6	1.31	0.001	− 1.5 ± 0.8	− 0.5 ± 0.7	1.30	0.001
Total	− 4.3 ± 1.8	− 1.9 ± 2.1	1.20	0.001	− 3.9 ± 2.4	− 1.6 ± 2.4	0.94	< 0.001

*t*0 pretreatment, *t*1 posttreatment (week 6), *t*2 follow-up (week 12).

*CSI* Central Sensitization Inventory, *VAS* visual analog scale, *WOMAC* Western Ontario and McMaster Universities Osteoarthritis Index

In terms of the regression analysis, the first model revealed that the delta change in VAS pain (during activity) after the treatment significantly affected central sensitization (*p* = 0.004, adjusted R^2^: 0.346). Change in VAS pain (during activity) from baseline to follow-up had also a significant effect on CSI scores (*p* = 0.002 adjusted *R*^2^: 0.391). In Model 2, delta changes in WOMAC pain from baseline to follow-up (*p* = 0.020, Δ*R*^2^ = 0.245) had a significant effect on CSI. However, Y Balance Test (both in the right and left leg), WOMAC physical function, and WOMAC total scores did not appear to have a significant impact on the improvement in central sensitization (*p* > 0.05). In addition, looking at the R^2^ change values, it can be concluded that changes in activity VAS pain alone could explain most of the change in CSI scores both after treatment and at follow-up (Table 4).

**Table 4:** Two model multiple linear regression analyses showing the effect of changes in VAS pain during activity, *Y* Balance Test, WOMAC pain, WOMAC physical function, and WOMAC total scores on the improvement in central sensitization

	Δ CSI score (t1–t0)			ΔCSI score (t2–t0)		
	*B* (SE)	*ß*	*p*	B(SE)	*ß*	*p*
Model 1						
ΔVAS pain during activity	2.1 (0.6)	0.6	0.004	3.9 (0.8)	0.8	0.002
Model 2						
Y Balance Test						
ΔLeft leg total score	0.1 (1.1)	0.1	0.490	− 1.3 (0.9)	− 0.5	0.167
ΔRight leg total score	− 1.3 (1.1)	− 0.4	0.299	− 0.3 (0.8)	− 0.1	0.707
WOMAC						
ΔPain	1.1 (1.6)	0.2	0.070	2.2 (1.7)	0.6	0.020
ΔPhysical function	0.6 (1.6)	0.1	0.369	3.7 (1.4)	0.8	0.206
ΔTotal score	0.3 (0.7)	0.1	0.730	0.2 (0.1)	0.5	0.525
Note	*R*^2^ = 0.346 for model 1			*R*^2^ = 0.391 for model 1		
	Δ*R*^2^ = 0.216 for model 2			Δ*R*^2^ = 0.245 for model 2		

*CSI *Central Sensitization Inventory, *B* unstandardized coefficients, *SE* standard error, *β* standardized coefficients, *R*^2^ adjusted *R*-squared, *WOMAC* Western Ontario and McMaster Universities Arthritis Index

## Discussion

Among patients with central sensitization due to OA, those who received a balance exercise program for 6 weeks experienced greater improvement in central sensitization, activity pain, dynamic balance, and knee functions compared to the control group. The beneficial effects of the exercise program lasted at least 6 weeks. However, there was no significant improvement in functional balance.

Central sensitization is common among patients with knee OA [30]. Non-pharmacological treatment methods are widely used in the management of knee OA [31–33]. Therapeutic exercises may reduce pain or hypersensitivity in OA [34–36]. Strengthening and aerobic exercises were shown to decrease pain sensitivity in patients with knee OA. Sensitivity to pain was evaluated by looking at the changes in pressure pain threshold or temporal pain summation [34, 35]. The decrease in pain sensitivity was determined only in the knee and surrounding structures. Moreover, hypersensitivity in more distant segments was not evaluated. Therefore, those results do not provide conclusive evidence regarding a potential decrease in central sensitization [9]. In the current study, balance exercises provided a decrease in central sensitization among patients with OA and central sensitization was evaluated by CSI, which enabled a global assessment. The beneficial effects could be attributed to the increase in proprioceptive inputs with balance exercises, which in turn might have led to a blockage in repetitive nociceptive stimuli [13] and a decrease in pain hypersensitivity. To clarify this, further regression analysis was performed. The results showed that, following a balance exercise program, the reduction in activity pain alone seemed to be a sufficient reason for the improvement in central sensitization. However, the improvement in balance did not directly affect central sensitization. Time-based exercises are recommended in patients who develop central sensitization due to OA [8]. Without any tissue damage, pain and warning signs are generated by the brain. Mild and moderate pain, particularly during exercise, is thought to be virtual pain created by the brain. In the time-dependent approach, the brain is prevented from processing the mechanisms that facilitate pain [8]. As recommended, a time-dependent approach was adopted in the current study. The patients were asked to continue the exercises even if they felt mild-to-moderate pain while performing the exercises. Adopting such an approach might have enabled us to obtain beneficial effects by exercise. At the end of the treatment program, the exercise group achieved a significant improvement in pain during activity, compared to the control group. Yet, such improvement was not observed in pain at rest. This discrepancy may be related to the potential selective effects of balance exercises on the proprioceptors involved during activity [37]. Damage to mechanoreceptors due to the degenerative changes in the knee joint and loss of quadriceps muscle strength can deteriorate balance and proprioception [13, 38]. Recent studies have shown that neuromuscular and balance exercises can significantly improve balance among patients with knee OA [15, 38]. The current study detected an improvement in dynamic balance after a 6-week balance exercise regimen. However, no significant difference was found in the functional balance, particularly during daily living activities. Patients with knee OA have difficulty in many functional activities, such as walking, climbing stairs, and standing up from sitting. Messier et al. reported that functional insufficiency may develop due to pain and decreased lower extremity muscle strength in older patients with OA [18]. There are conflicting results in the literature regarding the effects of balance exercises on pain and physical function in knee OA [16, 39]. In the current study, we found a clinically significant improvement in functionality with balance exercises. The improvement in pain and dynamic balance might be associated with the positive effects of the exercise program on functionality. However, we assessed function by the WOMAC questionnaire and did not use objective test batteries. It would be beneficial to support these findings with studies using objective tests to assess function. 

There are some limitations to be discussed in the current study. Although the CSI is a reliable questionnaire for evaluating central sensitization in many health conditions, it could be supported by other objective measures such as pressure pain threshold or temporal pain summation assessment. The second limitation of the study is that this study had a short-term follow-up. The long-term effects of the exercises on balance and central sensitization could not be determined. Another limitation is the lack of data regarding the participants’ regular exercise habits and nutritional status. Lastly, the patients were not blind to the treatment and a placebo group could not be formed due to the nature of the exercise regimen. 

In conclusion, a 6-week balance exercise program may improve central sensitization, pain during activity, dynamic balance, and function in patients with knee OA. In patients with centrally sensitized knee OA, balance exercises can be used to improve functional status, dynamic balance, and central sensitization. We recommend future studies based on promising avenues for the treatment of chronic OA-related central sensitization and including objective assessment methods and long-term follow-up to compare with the results of the current study.

## Data Availability

The datasets gathered during the preparation of this manuscript are available from the corresponding author upon reasonable request.
